# Heritability in the Efficiency of Nonsense-Mediated mRNA Decay in Humans

**DOI:** 10.1371/journal.pone.0011657

**Published:** 2010-07-21

**Authors:** Cathal Seoighe, Chris Gehring

**Affiliations:** 1 School of Mathematics, Statistics and Applied Mathematics, National University of Ireland Galway, Galway, Ireland; 2 Institute of Infectious Disease and Molecular Medicine, University of Cape Town, Cape Town, South Africa; 3 Computational Bioscience Research Centre, King Abdullah University of Science and Technology, Thuwal, Kingdom of Saudi Arabia; Centre de Regulació Genòmica, Spain

## Abstract

**Background:**

In eukaryotes mRNA transcripts of protein-coding genes in which an intron has been retained in the coding region normally result in premature stop codons and are therefore degraded through the nonsense-mediated mRNA decay (NMD) pathway. There is evidence in the form of selective pressure for in-frame stop codons in introns and a depletion of length three introns that this is an important and conserved quality-control mechanism. Yet recent reports have revealed that the efficiency of NMD varies across tissues and between individuals, with important clinical consequences.

**Principal Findings:**

Using previously published Affymetrix exon microarray data from cell lines genotyped as part of the International HapMap project, we investigated whether there are heritable, inter-individual differences in the abundance of intron-containing transcripts, potentially reflecting differences in the efficiency of NMD. We identified intronic probesets using EST data and report evidence of heritability in the extent of intron expression in 56 HapMap trios. We also used a genome-wide association approach to identify genetic markers associated with intron expression. Among the top candidates was a SNP in the *DCP1A* gene, which forms part of the decapping complex, involved in NMD.

**Conclusions:**

While we caution that some of the apparent inter-individual difference in intron expression may be attributable to different handling or treatments of cell lines, we hypothesize that there is significant polymorphism in the process of NMD, resulting in heritable differences in the abundance of intronic mRNA. Part of this phenotype is likely to be due to a polymorphism in a decapping enzyme on human chromosome 3.

## Introduction

The transcriptome of higher eukaryotes is complex and diverse, with multiple isoforms present for most genes, resulting from heterogeneity at several stages of the generation and processing of RNA from transcription initiation to splicing and polyadenylation. The diversity of the splicing step has been intensively studied. Microarrays that target splice junctions were used to demonstrate that the majority of human genes are alternatively spliced [1] and, more recently, next generation sequencing has provided even greater resolution [2], [3] such that alternatively spliced isoforms have now been observed from almost all human multi-exon genes. In addition to diversity across tissues, transcript isoforms show diversity between individuals, with about 20% of alternatively spliced genes showing evidence of inter-individual differences in relative isoform abundance [2], [4]. Mutations that affect splicing appear to be responsible for a large proportion of human genetic diseases (see [5], [6] for reviews) and may even be the largest contributor to human genetic diseases resulting from single point mutations [7]. Researchers investigating the potential functional implications of genetic variants have often tended to ignore splicing effects [5], although, recently, there have been several large-scale studies to identify common genetic variants with an effect on mRNA splicing [4], [8], [9], [10], [11], [12], [13], [14], [15]. These studies parallel efforts to determine the genetic contribution to gene expression variation (for review see [16]).

The rate at which mRNA of a transcript comes into the system is just one part of the equation determining mRNA levels. Different mRNA isoforms have different stabilities and can have decay rates ranging over orders of magnitude [17], [18]. This difference can be important for the regulation of gene expression levels. For example, steady state expression levels of genes with faster decay rates are altered more rapidly by changes in the rate of transcription than genes with slower decay rates [18]. While for most housekeeping genes decay rates are relatively constant, in some cases decay rates are regulated by proteins that bind to mRNA, with functional implications, for example in regulation of immunity [19]. Decay of mRNA requires removal of the 5′ 7-methylguanosine cap structure which is achieved by a decapping complex [20]. This occurs following deadenylation in deadenylation-dependent mRNA decay but is also required in nonsense-mediated decay (NMD) [20]. The NMD pathway degrades mRNAs with premature stop codons and acts as a surveillance step for incorrectly spliced or mutated transcripts. When coupled to regulated alternative splicing, it can be used as a means of post-transcriptional regulation [21]. More generally, NMD appears to be a key quality control step, conserved across eukaryotes, that prevents translation of incompletely spliced transcripts [22].

There is good evidence of variability in the efficiency of NMD across tissues [23], [24] and between individuals [25], [26]. This difference can have phenotypic and clinical implications [27], for example in the severity of genetic diseases resulting from premature termination codon (PTC) mutations [28] and in individual-specific responses to drugs [29]. Tissue-specific differences in NMD can result in PTC mutations with a phenotype in one tissue and not another. For example, a heterozygous PTC in the gene for collagen X causes Schmid metaphyseal chondrodysplasia [24]. The phenotype is restricted to cartilage, where NMD removes the PTC-containing transcripts, and the gene is not subject to NMD in other cell types. A difference in the efficiency of NMD has previously been reported to represent a stable phenotype in human cell lines that can result from differences in the abundance of NMD co-factors, possibly among other causes [26]. In general, because degrading improperly spliced mRNA from which introns have not been removed appears to be an important role of NMD in eukaryotes [22] it is reasonable to consider that there may be stable and heritable differences in the proportion of undegraded intron-containing transcripts.

In this study, we set out to determine whether there is evidence of transcriptome-wide differences in the abundance of intron-containing mRNA isoforms observed in different cell lines, using exon microarray data from published studies. We identified putative intronic probesets on the Affymetrix Human Exon 1.0 ST microarray and estimated the relative frequency with which these probesets were included in the final transcript. Intron-containing transcripts may be unprocessed, partially processed or fully processed mRNA in which one or more introns have been retained. Differences in the abundance of intron-containing transcripts may, therefore, reflect differences in the efficiency of splicing or in the efficiency with which intron-containing transcripts are degraded through NMD.

The microarray data included 56 complete trios from the HapMap [30] cell line panels originating from the Yoruba in Ibadan, Nigeria (YRI) and from Utah residents with ancestry from northern and western Europe (CEU). We calculated the normalized expression intensity of intronic probesets and report that the extent of intron expression shows evidence of heritability in the cell lines from the HapMap trios. We then performed a genome-wide association test, using parents of the HapMap trios to identify loci that were associated with normalized expression intensity of intronic probesets. One of the most significant peaks in the results of this test was within *DCP1A* on chromosome 3. *DCP1A* encodes a component of the decapping complex which is involved in NMD [31], [32]. A polymorphism at this locus was significantly associated with higher relative expression intensity of intronic probesets.

## Results and Discussion

We used data generated with the Affymetrix Human Exon 1.0 ST array from human cell lines to investigate heritability in the relative expression of introns in human. Because the probes on the array target computationally predicted exons as well as known exons and exons derived from ESTs and other sources, the targeted regions are likely to include a proportion of introns as well as correctly predicted exons. From among the approximately 1.4 million probesets on the array, we identified 269,007 that are likely to lie within introns, as described in Methods. Raw intensity (.CEL) data generated using this array by Huang *et al.* [33] was downloaded from GEO and processed as described in the Methods section. For each of these putative intronic probesets we estimated the normalized intensity as the expression intensity of the probeset divided by the metaprobeset-level summary value (i.e. transcript-level expression intensity). Interestingly, the normalized intensity of these intronic probesets was weakly, though highly significantly negatively correlated (Spearman ρ = −0.14; p<1×10^−16^) with the half-life of the transcript to which they mapped (mRNA half-lives estimated in human B cells were obtained from a recent study [34]). This is consistent with expectations because intron-containing transcripts are frequently subject to degradation (e.g. through NMD) and for long-lived transcripts, the fraction of the transcript pool corresponding to intron-containing but as yet undegraded transcripts should be lower than for transcripts with a higher turn over rate.

To investigate the possibility of transcriptome-wide differences in the proportion of intron containing transcripts, we restricted to those intronic probesets that were detectable above background (p<0.05) in at least 20 of the 176 HapMap individuals and for which at least one core probeset in the corresponding gene was detectable above background in the majority of the individuals. We then ranked the normalized intensities for the probeset across all of the arrays and obtained a summary statistic for each array by calculating the average rank of its normalized intensity values across all intronic probesets. This intron expression summary statistic showed substantial variability across arrays – indicating that in some of the cell lines the normalized intensity of intronic probesets (i.e. their relative inclusion in the mature transcript) is generally lower than for other cell lines. Values ranged from 56.6 (indicating an array for which, on average, the normalized intensity of intronic probesets ranked 56.6^th^ out of 176 arrays, i.e. in the lower 32.2%) to 119.4 (corresponding to the top 67.8%) for the array with the highest value. The distribution of the summary statistic across arrays is provided as supplementary figure S1.

**Figure 1:**
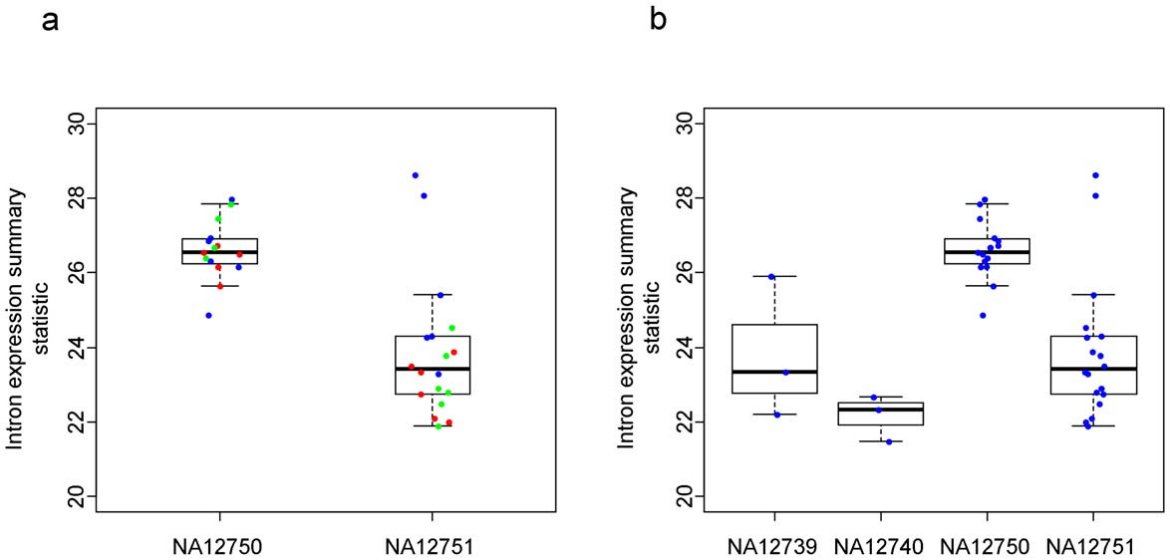
Reproducibility of the summary statistic of intron expression across replicate samples from a previous study. a) stripchart showing technical and biological replicates for two lymphoblast cell lines. Separate cell passages are shown in different colours. b) boxplots and superimposed stripcharts of biological replicates (separate cell passages) of four cell lines.

Although there was evidence of substantial difference in relative intron expression across arrays, it was not clear to what extent this reflected biologically significant differences between the samples or merely differences in the arrays or sample preparations. To investigate this we obtained a second set of data, consisting of exon microarrays applied to members of the CEPH 1444 pedigree by Kwan *et al.* [11]. In this case exon microarray data were available for three separate passages for four individuals as well as single passages for a further 10 individuals. The data were analyzed as described above. Five technical replicates for each of the three biological replicates (separate cell passages) were available for two of the cell lines (NA12750 and NA12751). Values of the intron expression statistic calculated from these arrays suggest that technical variation as well as variation between cell passages make a contribution to the observed differences between arrays (Fig. 1a); nonetheless, the difference between cell lines was highly statistically significant, despite the variation between repeats (p = 1.0×10^−5^, from a two-tailed t-test). Other than three of the technical replicates of the first passage of NA12751, the two cell lines were easily distinguishable. When we considered the data from all cell lines for which biological replicates were available we again found a significant difference across cell lines (Fig. 1b; p = 5.7×10^−6^ from a one-way ANOVA).

**Figure 2:**
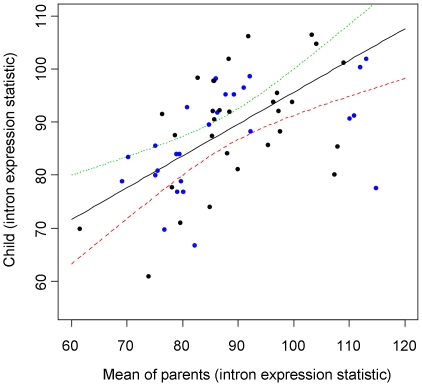
Heritability of the mean expression intensity of intronic probes. Points in the scatter plot of offspring values of the summary statistic against mean of the parent values are colored in blue for CEU trios and black for YRI trios. The estimated regression line (from the combined data) together with upper (green) and lower (red) bounds on the regression line estimates are shown.

To search for evidence of heritability in relative intron expression we returned to the exon array data from the HapMap trios [30] and regressed the summary statistic obtained from the offspring against the mean value of the parents (Fig. 2). This suggests strong heritability of the relative intron expression (slope = 0.60±0.28; p = 7.5×10^−5^). There was evidence of heritability, considering the CEU and YRI samples separately (p = 0.01 and p = 0.003 from the regressions of CEU and YRI trios, respectively), providing an indication of the robustness of the result to sampling and batch effects, especially as these samples were collected decades apart [35]. Batch effects have been shown to be a significant concern in microarray experiments [36] and this study could be prone to such effects, given the observed variability across biological and technical repeats (Fig. 1). Although, the authors of the study from which the microarray data were obtained took care to avoid batch effects [33], we also extracted date information from the headers of the CEL files and found that there was some evidence, albeit very weak, of members of the same trio having similar processing dates (p = 0.05 from one-way ANOVA of sampling times against trio membership). However, this seems unlikely to be responsible for the strong heritability evident in figure 2.

As an additional check, we also compared the intron expression statistic between parents of the trios and found that these were significantly correlated (r = 0.44; p = 0.0007), suggesting that a batch effect may be involved in the apparent heritability of the intron expression summary statistic. When we analyzed the CEU and YRI samples separately we found that the correlation between parents was significant only for the YRI (p = 0.0003 and p = 0.23 for the YRI and CEU trios, respectively). The YRI parents have been found to be more closely related to one another than random pairs of YRI samples while this was not the case for the parents of the CEU trios [37]. But this is a relatively small effect (the parents are not close relatives) and it therefore appears likely that there is a non-genetic contribution to the similarity observed between the YRI parents. However, there is no evidence of such an effect in the CEU samples and the CEU and YRI trios provide similar estimates of the heritability of the intron expression statistic (the slope of the regression line is 0.69 with a standard error of 0.25 for the CEU trios and 0.53 with a standard error of 0.16 for the YRI).

**Figure 3:**
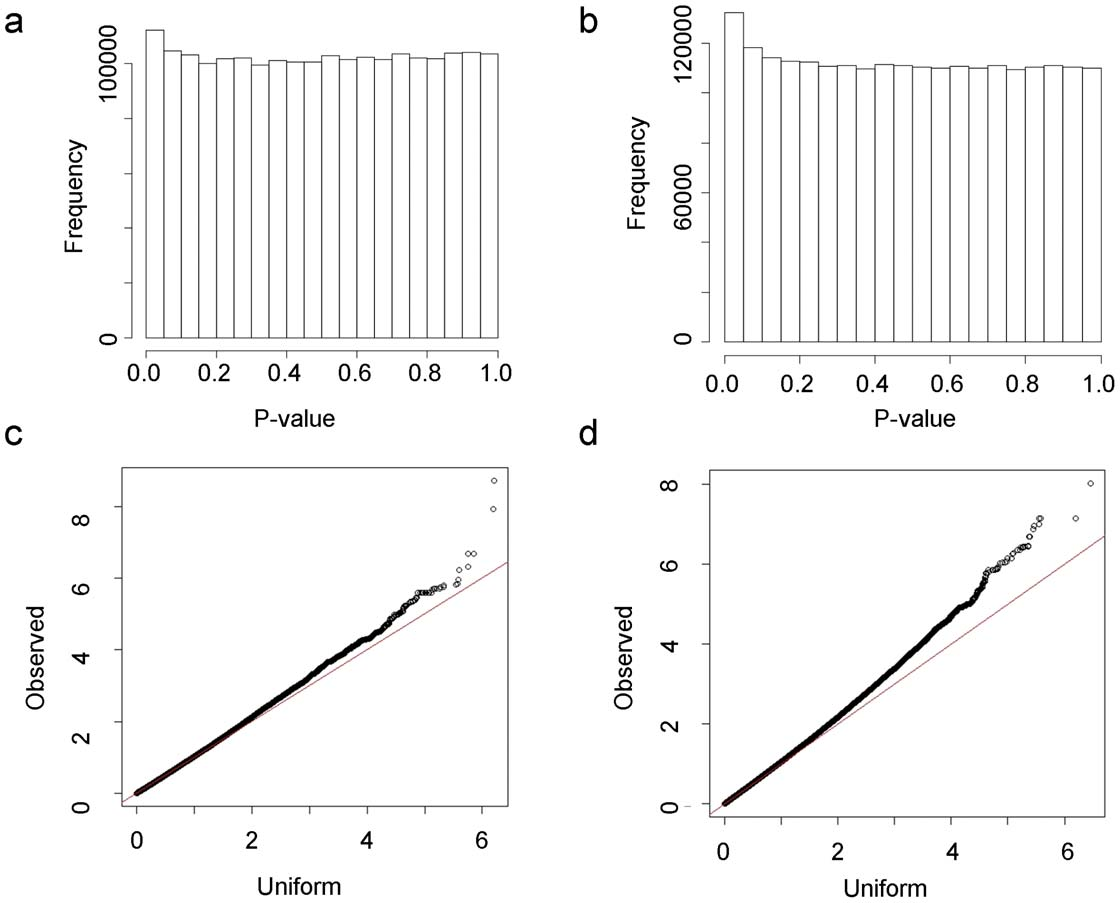
Histograms of p-values from tests of association between individual SNP markers and the intron expression phenotype in the CEU (a) and YRI (b) populations. Quantile-quantile plots of log_10_ of the p-values from the CEU (c) and YRI (d) association tests against log_10_ of random values, drawn from the uniform distribution.

**Figure 4:**
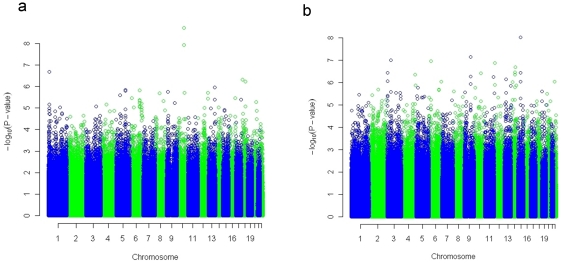
Genomic distribution of p-values from association tests. Successive chromosomes are shown in alternating colours on the plot. Results from the CEU and YRI populations are shown in panels a and b, respectively.

We used a genome-wide association approach to search for loci that are associated with the relative intron expression statistic. To avoid the inclusion of close relatives, we took only the data from the parents of the HapMap trios and carried out an additive test of association between SNPs genotyped as part of the HapMap project and the intron expression summary statistic. We carried out the test separately on the CEU and YRI samples, to avoid the effects of this population structure on the analysis. Histograms of the distributions of p-values obtained from individual tests of association and qqplots of the logarithm to the base ten of the p values against the logarithm of random draws from the uniform distribution are shown in figure 3 for both populations. In these plots it is clear that there were more low p values than expected under the uniform distribution, particularly in the YRI case, pointing to a proportion of true positive associations between the phenotype and a subset of the genetic markers. We used the qvalue package [38], [39] to estimate false discovery rates, bearing in mind the caveat that linkage between SNPs results in non-independent hypothesis tests. There were 45 markers with q values less 0.05. These 45 markers occurred in 19 peaks of the genomic plot of p values (Fig. 4).

**Figure 5:**
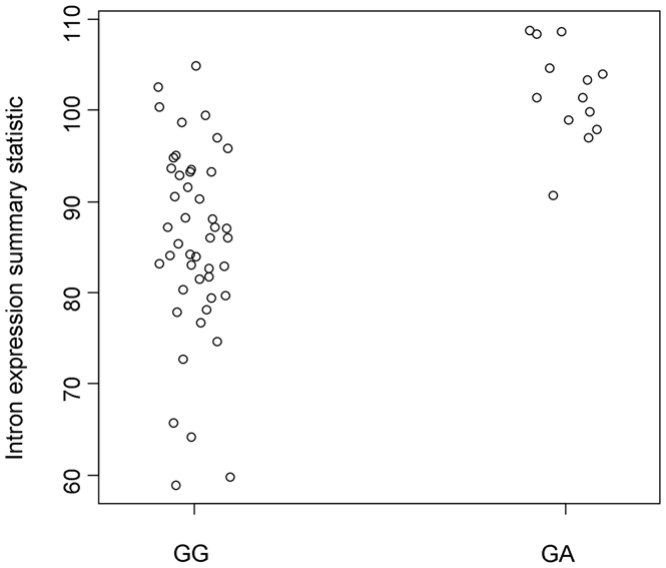
Stripchart of the relative intron expression phenotype against the genotype of SNP rs9311496 in the YRI population.

Markers associated with the intron expression phenotype are shown in Table 1. Of the 11 association peaks, four mapped within or close to genes. The most significant peak was within the NRG3 gene on chromosome 10, which was associated with intron expression in the CEU population. Although there is no obvious association between NRG3 and RNA processing it is interesting that a strong association to the same region of chromosome 10 was also observed in the YRI population (Table 1). The most promising of the associations was to a region of chromosome 3, within the *DCP1A* gene which encodes the human homologue of the yeast decapping enzyme, which, together with another decapping enzyme forms the human decapping complex, a key participant in deadenylation-dependent as well as nonsense-mediated mRNA decay. In yeast, the rate of decapping is thought to be a key determinant of the rate of mRNA decay and this gene was first identified through the effect of mutants on NMD [40]. NMD also involves decapping in higher eukaryotes [41]. The derived A allele of the G/A SNP which was associated with intron content occurs at a frequency of 11% in YRI but is not found in the non-African samples in HapMap II. Among the parents of the YRI trios, thirteen were heterozygous but there were no homozygous A individuals. The heterozygotes showed a greater normalized expression intensity of intronic probesets than the remainder of the parent samples (Fig. 5; p = 9.6×10^−8^).

**Table 1:** Markers associated with the intron expression phenotype with q-values<0.05.

SNP	Location	Function	Associated gene	Population	P-value
rs7088129	10: 84230104	Intronic	NRG3	CEU	1.9×10^−9^
rs659554	10: 84341085	Intronic	NRG3	CEU	1.2×10^−8^
rs2115904	15: 29424204	Intergenic	None	YRI	9.2×10^−9^
rs4878127	9: 89577039	Near 5′	CTSL3	YRI	7.1×10^−8^
rs17053466	9: 89579157	Intronic	CTSL3	YRI	7.1×10^−8^
rs10512189	9: 89580577	Intronic	CTSL3	YRI	7.1×10^−8^
rs9311496	3: 53347118	Intronic	DCP1A	YRI	9.6×10^−8^
rs16889633	6: 24811421	Intergenic	None	YRI	1.1×10^−7^
rs686394	12: 9966342	Intergenic	None	YRI	1.3×10^−7^
rs17101452	14: 74157738	Intergenic	None	YRI	2.0×10^−7^
rs888419	14: 74158848	Intergenic	None	YRI	2.0×10^−7^
rs4899337	14: 69740936	Intergenic	None	YRI	3.5×10^−7^
rs13329672	15: 56487229	Intergenic	None	YRI	3.5×10^−7^
rs410509	3: 5216309	Synonymous	EDEM1	YRI	3.5×10^−7^
rs377120	3: 5216223	Intronic	EDEM1	YRI	3.6×10^−9^
rs11201378	10: 86856283	Intergenic	None	YRI	3.7×10^−7^
rs12244919	10: 86860618	Intergenic	None	YRI	3.7×10^−7^
rs12252660	10: 86860718	Intergenic	None	YRI	3.7×10^−7^
rs11201382	10: 86862554	Intergenic	None	YRI	3.7×10^−7^

The table shows the combined results of the association test in the CEU and YRI populations.

As a further test for evidence of a contribution of NMD to the differences in the intron expression, observed between individuals, we identified a set of unprocessed pseudogenes, expressed above background (p<0.05) in at least 10 cell lines. Because NMD is involved in degrading pseudogenes [42] we hypothesized that the expression level of pseudogenes might be correlated with the intron expression statistic and show differences between individuals with different genotypes at the *DCP1A* locus. For each metaprobeset corresponding to one of the unprocessed pseudogenes we calculated the Spearman correlation coefficient between the metagene expression level and the intron expression statistic and compared these numbers to correlation coefficients obtained for non-pseudogenes. The Spearman rho statistics obtained from the processed pseudogenes were higher than for other genes (p = 0.003; Wilcoxon rank-sum test). For three of the 16 pseudogenes the correlation between expression level and the intron expression statistic was nominally significant (α = 0.05). Similarly, the expression levels of the pseudogenes were much more likely to be affected by the genotype at the *DCP1A* locus than other genes (p = 0.006, based on a t-test of t-statistics from 2-sample t-tests). In the case of four pseudogenes the differences in expression levels of were nominally significant (α = 0.05), and, in each case the pseudogene was more highly expressed in heterozygotes than homozygotes, as expected.

In conclusion, we found evidence that the normalized expression intensity, averaged across intronic probesets, shows reproducible differences between individuals and report that this appears to be a heritable trait in humans. However, we caution that this analysis is subject to any batch effects relating to the collection and treatment of the cell lines and report a correlation between parents of the YRI HapMap trios that we were not able to explain fully. This is the first study to explore transcript-wide differences between human individuals in the types of mRNA isoforms observed and it points to the contribution of *trans*-acting factors to the diversity of the transcriptome. To investigate what some of these *trans* factors might be we carried out a genome-wide association test of this phenotype using the densely genotyped HapMap cell lines and found several association peaks, including a strong association with one very plausible candidate gene – *DCP1A*, a component of the mRNA decapping complex. We propose that the derived allele of this SNP, which occurs at about 11% frequency in YRI and was not found in the CEU samples, is linked to a less efficient form of the decapping enzyme, resulting in a greater proportion of undegraded intron-containing mRNA.

## Methods

### Data

Raw microarray intensity data generated using the Affymetrix GeneChip Human Exon 1.0 ST array from 176 HapMap cell lines by Huang *et al.* [33] and from 14 cell lines from CEPH pedigree 1444 by Kwan *et al.* [11] were obtained from GEO and processed using the Affymetrix Power Tools as described previously [8]. Publicly available genotype data for almost four million markers were downloaded from the HapMap [30]. BLAT [43] alignments of EST sequences to the human genome were downloaded from UCSC (hg18, corresponding to NCBI genome build 36.1) on 17/3/2008. Only ESTs that mapped to the genome exactly once, with at least 95% identity over at least 90% of the length of the EST sequence were considered. mRNA half-life data in human B cells were obtained from a recent study [34].

### Identification of intronic probesets

For each probeset from the exon array, we counted the number of times the probeset was within the spliced portion of an EST, thus putatively in an intron. Spliced portions of ESTs were inferred from gaps in the alignments of ESTs to the genome, of at least 50 bp, surrounded by upstream and downstream aligned blocks of at least 20 bp. We also counted the number of times the probeset fell within the exonic (i.e. aligned) portion of an EST. Probesets that occurred in intron-like gaps in the genomic alignments of at least 10 ESTs and which occurred at least five times as frequently in gaps than in aligned blocks were designated as intronic. In some cases, these probesets may overlap skipped exons that are included infrequently in the mature transcript. However, given the large number of probesets identified in intron-like alignment gaps (269,0007) and given the much greater length of introns than exons, the majority of these probesets are likely to be non-exonic.

### Summary statistic of relative intron expression

We calculated normalized probeset intensity by dividing the probeset intensity by the estimated intensity of the corresponding meta-probeset (i.e. transcript) [44]. For each intronic probeset, identified as described above, we compared the normalized intensity to the normalized intensity of the same probeset in the other cell lines in order to obtain a rank ordering of the normalized intensities for the probeset across cell lines. This gives an indication of how frequently the intronic probeset is retained in the mature transcript, in a given cell line, compared to the other cell lines. This quantity was averaged across all probesets to obtain a summary statistic of the relative expression intensity of intronic probesets. The heritability of this quantity was investigated by regressing the child values against the mean of the parent values in HapMap trios for which microarray data were available for the offspring and both parents (56 trios).

### Genome-wide association test

We obtained genotype data from the CEU and YRI trios from HapMap [30]. To remove obvious close relatives we discarded the child data and considered only the parents of the trios. To avoid the effects of population structure between the YRI and CEU, we performed the test of association separately for each population group. To reduce the size of the multiple hypothesis testing problem we removed markers with fewer than 5 heterozygotes. We also considered only autosomal markers. We used the R package GenABEL [45] to perform additive tests of association (i.e. a linear model of the phenotype against the number of copies of the non-reference allele – zero for reference allele homozygotes, one for heterozygotes and two for non-reference allele homozygotes). We used the qvalue package within R for multiple test correction and investigated peaks in the p value distribution with q values below 0.25.

## Supporting Information

Figure S1 Distribution of the summary statistic of intron expression across cell lines.
(1.32 MB TIF)
