# Supplementary figures and images for: Heritability in the Efficiency of Nonsense-Mediated mRNA Decay in Humans
Source: PLoS One. 2010 Jul 21;5(7):e11657. doi: 10.1371/journal.pone.0011657 (PMC2908117; doi:10.1371/journal.pone.0011657)

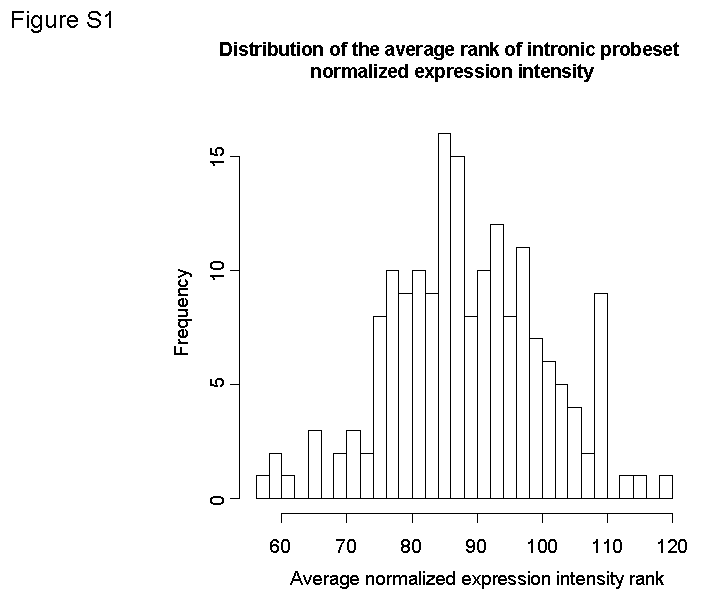

Supplement: Figure S1 — Distribution of the summary statistic of intron expression across cell lines. (1.32 MB TIF) [file pone.0011657.s001.tif]
